# Tumor spatial heterogeneity in myxoid-containing soft tissue using texture analysis of diffusion-weighted MRI

**DOI:** 10.1371/journal.pone.0181339

**Published:** 2017-07-14

**Authors:** Hyun Su Kim, Jae-Hun Kim, Young Cheol Yoon, Bong Keun Choe

**Affiliations:** 1 Department of Radiology, Samsung Medical Center, Sungkyunkwan University School of Medicine, #50 Irwon-dong, Gangnam-gu, Seoul, Republic of Korea; 2 Department of Preventive Medicine, Medical College, Kyung Hee University, Seoul, Republic of Korea; Suzhou University, CHINA

## Abstract

The objective of this study was to examine the tumor spatial heterogeneity in myxoid-containing soft-tissue tumors (STTs) using texture analysis of diffusion-weighted imaging (DWI). A total of 40 patients with myxoid-containing STTs (23 benign and 17 malignant) were included in this study. The region of interest (ROI) was manually drawn on the apparent diffusion coefficient (ADC) map. For texture analysis, the global (mean, standard deviation, skewness, and kurtosis), regional (intensity variability and size-zone variability), and local features (energy, entropy, correlation, contrast, homogeneity, variance, and maximum probability) were extracted from the ADC map. Student’s t-test was used to test the difference between group means. Analysis of covariance (ANCOVA) was performed with adjustments for age, sex, and tumor volume. The receiver operating characteristic (ROC) analysis was performed to compare diagnostic performances. Malignant myxoid-containing STTs had significantly higher kurtosis (*P* = 0.040), energy (*P* = 0.034), correlation (*P*<0.001), and homogeneity (*P* = 0.003), but significantly lower contrast (*P*<0.001) and variance (*P* = 0.001) compared with benign myxoid-containing STTs. Contrast showed the highest area under the curve (AUC = 0.923, *P*<0.001), sensitivity (94.12%), and specificity (86.96%). Our results reveal the potential utility of texture analysis of ADC maps for differentiating benign and malignant myxoid-containing STTs.

## Introduction

Myxoid-containing soft-tissue tumors (STTs) are an uncommon, heterogeneous group of mesenchymal neoplasms with variable histologic features. They are characterized by the presence of abundant extracellular myxoid matrix secreted by the tumor cells. Myxoid matrix, which has very high water content, results in distinctive imaging features on magnetic resonance imaging (MRI) with extremely low and high signal intensity on T1- and T2-weighted sequences, respectively [1]. When characterizing myxoid-containing STTs, there is considerable overlap in the signal characteristics between benign and malignant myxoid-containing STTs on the conventional T1- and T2-weighted MRI, which necessitates the use of biopsy for diagnostic confirmation.

In addition to conventional MRI, diffusion-weighted imaging (DWI) has recently been used to characterize soft tissue lesions. DWI can detect differences in the Brownian motion of water protons depending on variations in the tissue microstructure. For image quantification, the apparent diffusion coefficient (ADC) value, which has been widely studied as an imaging biomarker for tumor cellularity, can be estimated from at least two DWIs with different b values. It has been reported that DWI has the potential to differentiate benign and malignant STTs, but many discrepancies exist in the literature [2–8]. Van Rijswijk et al.[3], for example, reported that the mean ADC values of the malignant STTs were significantly lower than those of the benign STTs from 23 patients. Other authors [7] reported that there were no significant difference between the malignant and the benign STTs in their study of 29 patients, because of the overlap in the ADC values between the two groups. In a large study of 88 subjects, Nagata et al. [8] reported that mean ADC values for myxoid-containing STTs were significantly higher than those of their non-myxoid-containing counterparts, but they could not find significant difference in the mean ADC values between benign and malignant myxoid-containing STTs. According to the previous studies, the mean ADC value has limited ability to discriminate between benign and malignant myxoid-containing STTs since it can be affected by the extracellular matrix containing large amount of water as well as by tissue cellularity. Thus, more sophisticated parameter other than mean ADC value is required for differentiating benign and malignant myxoid-containing STTs.

Texture analysis is an emerging oncological imaging tool that provides information on intralesional heterogeneity without the need for additional image acquisition [9,10]. Significant heterogeneity exists within tumor due to regional variation in cell proliferation, cell death, metabolic activity, and vascular structure. Over the past few decades, numerous published articles have shown the ability of texture analysis to extract diagnostically valuable information from medical images obtained using various imaging modalities [11–14]. Many studies have reported promising results of texture analysis regarding diagnosis, grading, classification, and survival prediction of tumors in various organs [13–18]. Recently, encouraging results have emerged from preliminary studies on texture analysis of conventional MRI used to differentiate benign and malignant STTs [19,20]. Regarding texture analysis of DWI, there are a few studies which revealed potential value of these parameters as clinically relevant imaging marker in tumors of brain, breast, and ovaries [12,21,22].

To our knowledge, the usefulness of texture analysis of DWI in myxoid-containing STTs has not been fully examined. Little is known about the spatial heterogeneity in the myxoid-containing STTs. In addition, there are no published studies regarding texture analysis of DWI for differentiating benign and malignant myxoid-containing STTs. Thus, the purpose of this study was to examine the spatial heterogeneity in myxoid-containing STTs, and to investigate the utility of DWI texture analysis for differentiating benign and malignant myxoid-containing STTs.

## Materials and methods

### Study subjects

This retrospective study was approved by the Institutional Review Board of the Samsung Medical Center (IRB File No. 2015-12-023), which waived the requirement of informed consent. From June 2011 to June 2014, patients who were pathologically diagnosed with myxoid-containing STTs and underwent musculoskeletal MRI including DWI before the procedure were included in this study. Forty consecutive subjects (18 women, 22 men) with 40 myxoid-containing STTs (23 benign and 17 malignant myxoid-containing STTs) were included in the analyses. Six tumors, which were all benign, were pathologically confirmed by image-guided biopsy and 34 tumors were confirmed by surgical excision. The 23 benign myxoid-containing STTs consisted of schwannomas (n = 18), intramuscular myxomas (n = 3), and neurofibromas (n = 2) while the 17 malignant myxoid-containing STTs included myxoid liposarcomas (n = 10), myxofibrosarcomas (n = 3), malignant peripheral nerve sheath tumors (n = 2), and extraskeletal myxoid chondrosarcomas (n = 2).

### Magnetic resonance imaging protocol

All 40 patients were examined using a 3.0-T MRI system (Achieva TX; Philips Healthcare, Best, The Netherlands). Various radiofrequency (RF) coils and MRI parameters were used based on the anatomical location of the lesions (Table 1). A single-shot spin-echo echo-planar DWI sequence was performed in the axial plane. Sensitizing diffusion gradients were sequentially applied in the x, y, and z directions with *b* values of 0, 400, and 800 s/mm^2^.

**Table 1:** MRI parameters.

Parameter	Conventional Imaging	DW Imaging
Field of view (mm)	Variable	160 or 350
Matrix size	Variable	256×256
Repetition time (ms)/echo time (ms)	T1-weighted axial and coronal imaging: 400-520/15-16; T2-weighted axial and sagittal imaging: 2411-5366/80-100; fat-suppressed T1-weighted imaging: 441-561/15-16	5/61-69
Fat suppression	Chemical shift-selective pulse	Chemical shift-selective pulse
Section thickness (mm)	3–8	5
Intersection gap (mm)	0–1	
Turbo factor	Variable	
Echo train length	Variable	59–67
No. of signals acquired	Variable	2
Flip angle	0	90°

DW = diffusion-weighted, No. = number

### Image data analyses

**Fig 1:**
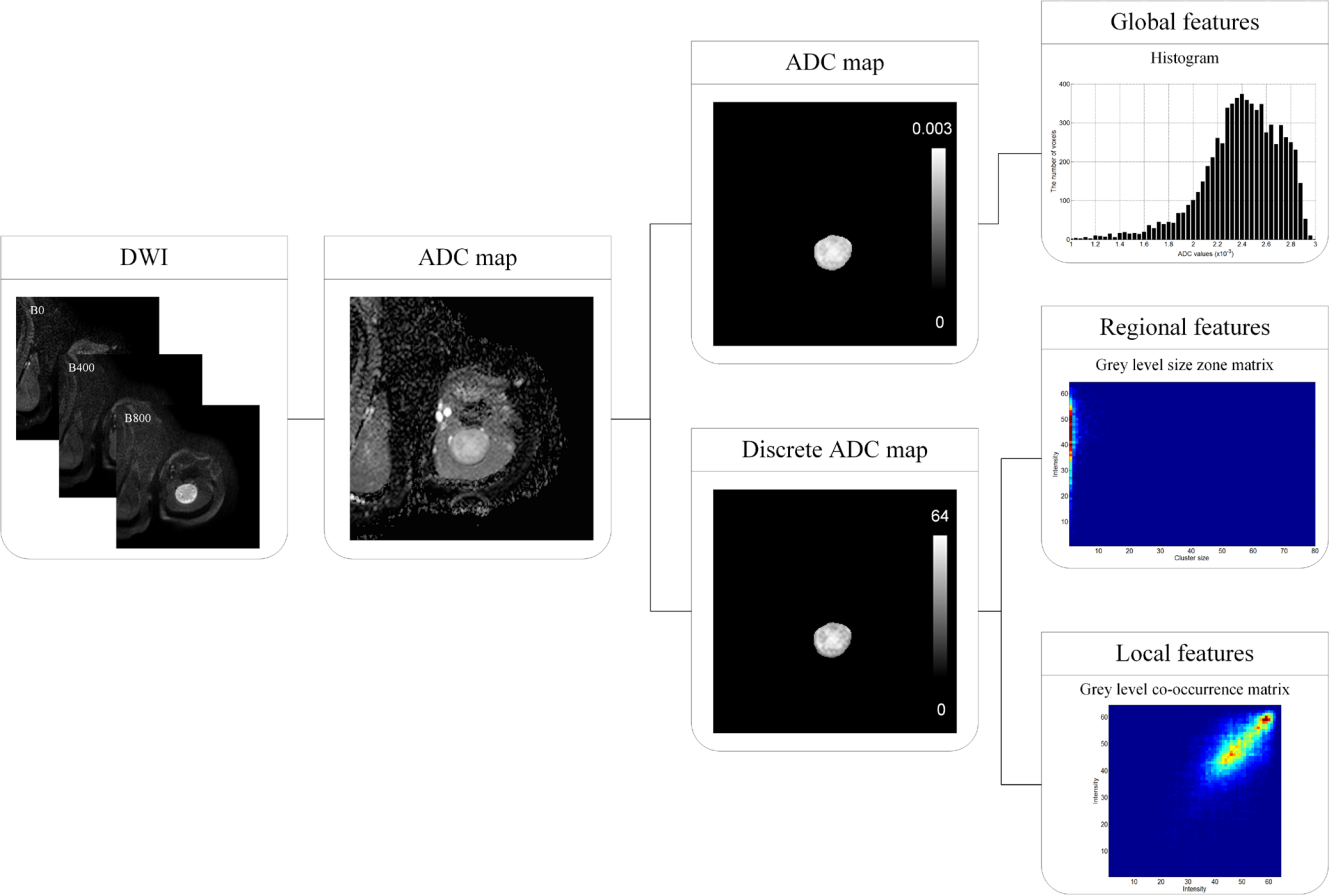
Flow chart for texture analysis of DWI in myxoid-containing STTs. For global features, a histogram was derived from the distribution of the voxels’ ADC values within the ROI. For regional and local texture analysis, the ADC values were resampled to yield 64 discrete values. For regional features, the grey level size zone matrix’s (m, n) was defined by the number of homogenous regions given the ratio of homogeneous tumor size (n) to the regions’ intensities (m). For local features, the grey level co-occurrence matrix (GLCM) was created.

For image analysis, all DWI data was transferred to a personal workstation and analyzed using in-house software written using MATLAB v. 7.6 (Mathworks, Natick, MA). Fig 1 shows the flow chart for image processing using DWI texture analysis in myxoid-containing STTs. ADC maps were computed by exponential fitting of the local signal intensities using three b values (0, 400, and 800 s/mm^2^). The region of interest (ROI) was manually drawn on the ADC map by a single radiologist (H.S.K) with 11 years of experience in musculoskeletal radiology who was blinded to radiologic reports, clinical information, and histopathologic results. ROIs were drawn on each section of the ADC maps in order to contain the entire tumor. The tumor volume was calculated by multiplying the number of voxels within the ROI by the unit volume of a voxel. Texture analysis was performed by a radiology physicist (J.H.K) with 7 years of experience in radiology physics. For global features, a histogram was derived from the distribution of the voxels’ ADC values within the ROI. From the histogram, we computed the mean (1^st^ statistical momentum), standard deviation (2^nd^ statistical momentum), skewness (3^rd^ statistical momentum), and kurtosis (4^th^ statistical momentum). For regional and local texture analysis, the ADC values within the ROI were resampled to yield 64 discrete values ranging from 1 to 64 (discrete ADC map). For regional features, the grey level size zone matrix’s (GLSZM’s) (m, n) was defined by the number of homogenous regions, given the homogeneous tumor size (n) and the intensity (m). From GLSZM, the intensity variability and size-zone variability were computed [23]. For local features, the grey level co-occurrence matrix (GLCM) was created for all 13 directions with a 1-voxel distance [24]. To create rotation-invariant GLCM, we averaged each GLCM direction, and from the averaged GLCM, 7 local features (energy, entropy, correlation, contrast, homogeneity, variance, and maximum probability) were computed. In summary, we extracted 4 global, 2 regional, and 7 local features from the ROI (Table 2).

**Table 2:** Thirteen texture features used to differentiate benign and malignant myxoid-containing soft tissue tumors.

Type	Texture Features
*Global Features Based on Histogram*	Mean
	Standard Deviation
	Skewness
	Kurtosis
Regional Features Based on Grey Level Size Zone Matrix (GLSZM)	Intensity Variability
	Size-zone Variability
Local Features Based on Grey Level Co-occurrence Matrix (GLCM)	Energy
	Entropy
	Correlation
	Contrast
	Homogeneity
	Variance
	Maximum Probability

### Statistical analyses

All statistical analyses were performed using SAS statistical software (version 9.4, SAS Institute, Cary, NC, USA) and MedCalc software (version 12.7.2, MedCalc, Mariakerke, Belgium). Normality of data distribution was assessed using the Kolmogorov-Smirnov test. Sex differences between the groups were tested by a Chi-square test. A student’s t-test was used to test the difference between group means. Analysis of covariance (ANCOVA) was performed with adjustments for age, sex, and tumor volume. For the texture parameters that showed significant difference between benign and malignant STTs in ANCOVA, a receiver operation characteristic (ROC) curve was constructed and the area under the curve (AUC) and the corresponding *P*-values were calculated. Optimal cutoff value was determined by maximizing the Youden index (J = sensitivity + specificity– 1) and 95% confidence interval (CI) was calculated using binomial exact. Sensitivity and specificity were calculated for the thus determined optimal cutoff values. The discriminatory power was classified as follows, based on AUC: 0.9–1 = excellent, 0.8–0.9 = good, 0.7–0.8 = fair, 0.6–0.7 = poor, 0.5–0.6 = failure. In addition, pairwise comparisons of AUC for all variables were performed and Bonferroni’s correction was used to adjust *P*-value for multiple comparisons. A *P-*value less than 0.05 was considered statistically significant.

## Results

The statistical significance of age, sex, tumor volume, and texture analysis parameters of ADCs between benign and malignant myxoid-containing STTs are summarized in Table 3.

**Table 3:** Comparison of tumor volume and texture analysis parameters between benign and malignant myxoid-containing soft tissue tumors.

Variable	Benign	Malignancy	Unadjusted	Adjusted
			*P*-value†	*P*-value‡
Subjects (F:M)	23 (12:11)	17 (6:11)	0.289*	
Age (year)	53.0±12.7	60.8±13.4	0.068	-
Volume (cm^3^)	15.630±14.803	139.355±179.174	0.009^§^	-
Global features				
Mean (10^−3^)	2.049±0.525	1.914±0.408	0.384	0.346
SD (10^−3^)	0.341±0.141	0.327±0.135	0.760	0.069
Skewness	-0.231±0.761	-0.341±0.876	0.672	0.645
Kurtosis	3.731±1.658	4.754±3.414	0.217	0.040^§^
Regional features				
Intensity Variability	138.315±81.126	391.686±423.172	0.026^§^	0.548
Size-zone Variability	5.312±2.773	17.569±17.396	0.011^§^	0.258
Local features				
Energy	0.003±0.002	0.004±0.003	0.287	0.034^§^
Entropy	2.644±0.255	2.629±0.246	0.854	0.151
Correlation	0.007±0.003	0.014±0.009	0.005^§^	<0.001^§^
Contrast	74.956±27.061	37.433±14.995	<0.001^§^	<0.001^§^
Homogeneity	0.271±0.046	0.337±0.057	<0.001^§^	0.003^§^
Variance	89.169±40.827	55.633±27.252	0.006^§^	0.001^§^
MP	0.010±0.005	0.011±0.007	0.567	0.096

Values are the mean ± standard deviation except where otherwise indicated. SD = standard deviation, MP = maximum probability

* Value was determined by Chi-square test.

† Values were determined by t-test.

‡ Values were determined after adjustment for age, sex, and tumor volume by using ANCOVA regression analysis.

^§^*P*<0.05, statistically significant.

### Population characteristics and tumor volume

**Fig 2:**
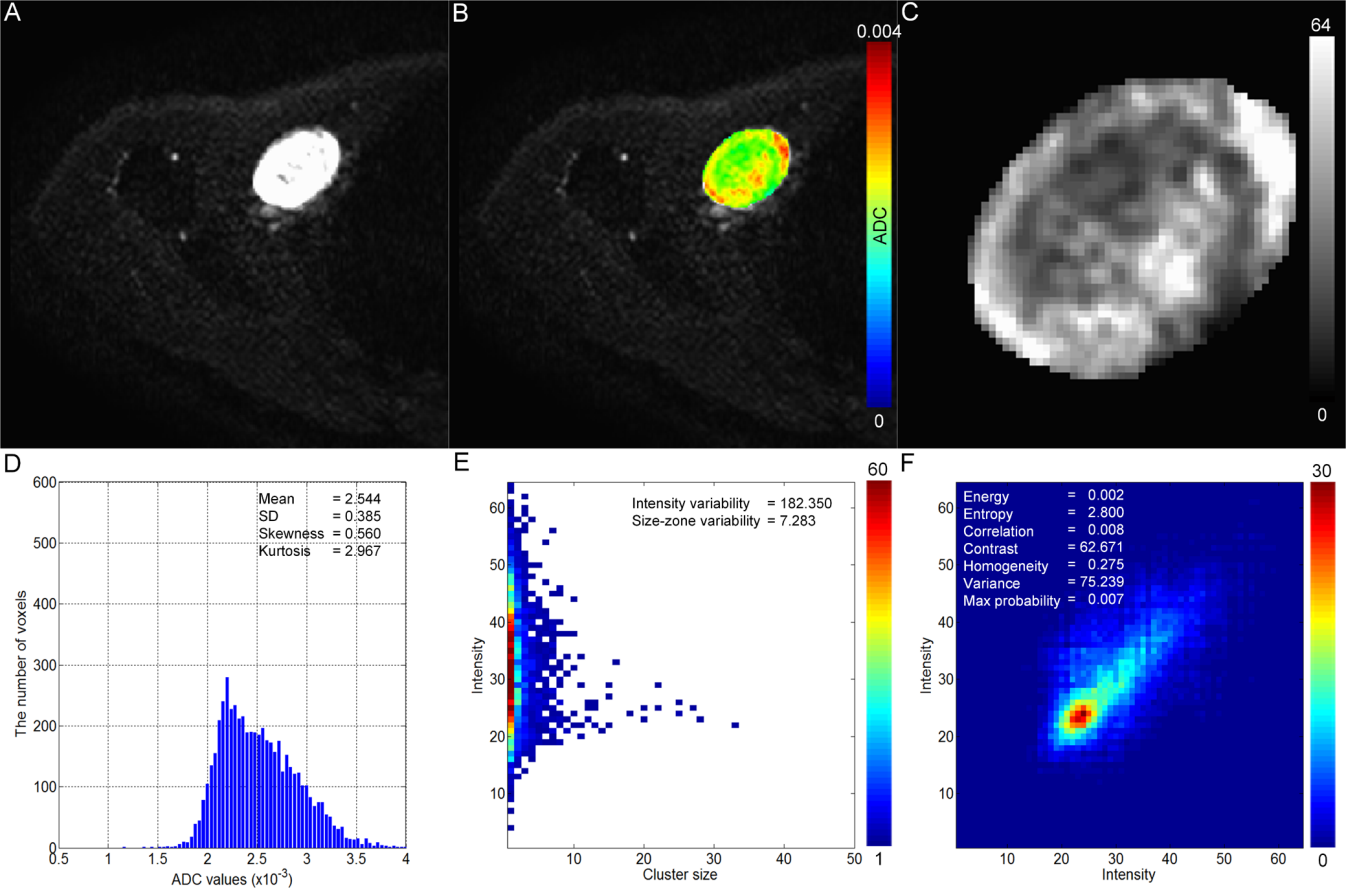
A case of benign myxoid-containing STT (schwannoma) in a 48-year-old female. DWI (A) shows a circumscribed high signal intensity mass in the right axillary region. Using a color ADC map (B), a discrete ADC map with 64 different values ranging from 1 to 64 (C), a histogram (D), a GLSZM (E), and a GLCM (F) were generated.

**Fig 3:**
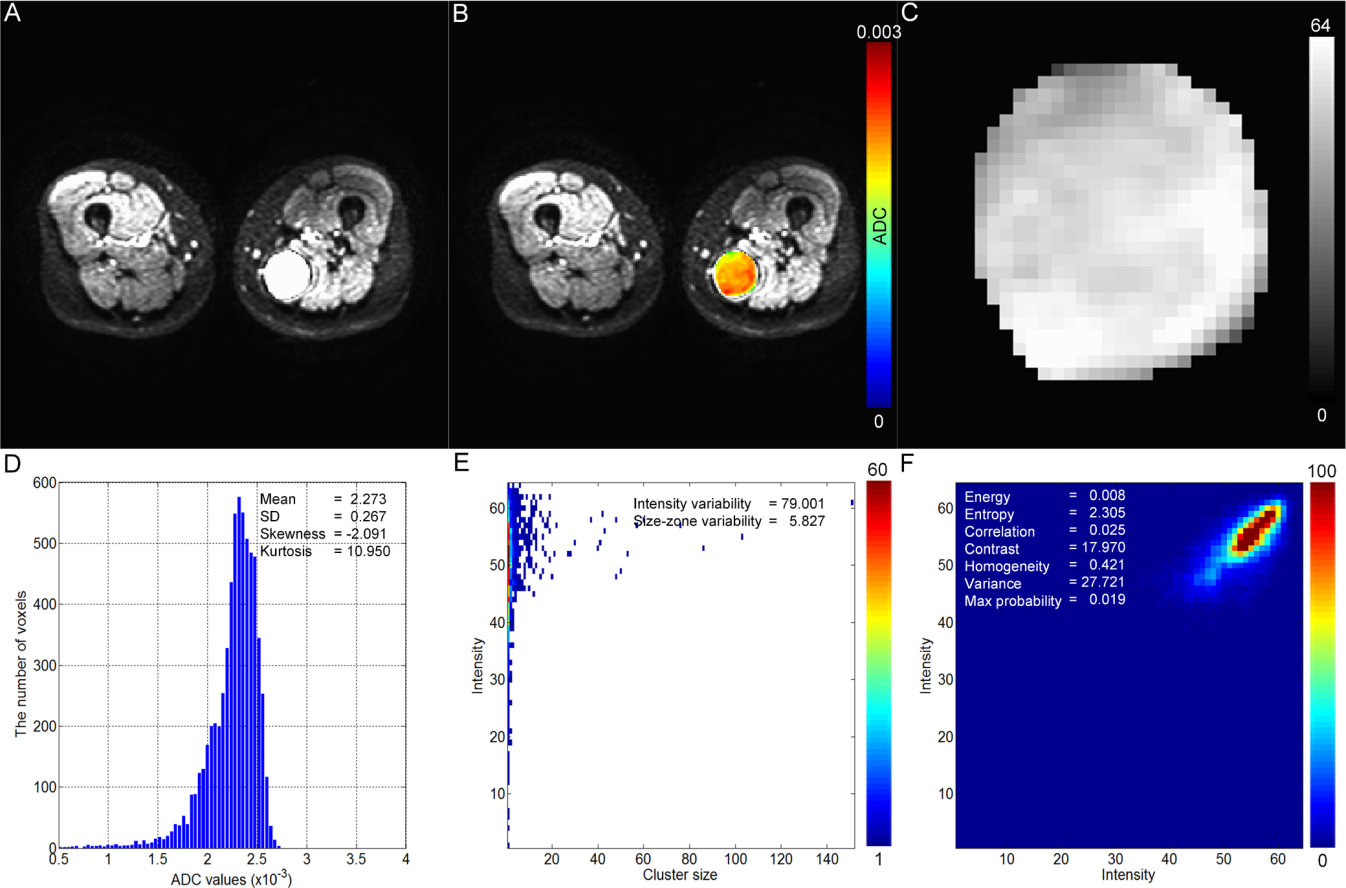
A case of malignant myxoid-containing STT (myxoid liposarcoma) in a 52-year-old female. DWI (A) shows a circumscribed high signal intensity mass in the left posteromedial thigh. Using a color ADC map (B), a discrete ADC map with 64 different values ranging from 1 to 64 (C), a histogram (D), a GLSZM (E), and a GLCM (F) were generated.

Sex ratios and ages were not significantly different between the two groups (*P* = 0.289 and 0.068); however the mean tumor volume of malignant myxoid-containing STTs was significantly higher than that of benign myxoid-containing STTs (*P* = 0.009). Fig 2 shows the DWI (a), ADC map (b), discrete 6-bit ADC map (c), histogram (d), GLSZM (e), and GLCM (f) of a representative benign myxoid-containing STT, while Fig 3 shows these parameters in a malignant myxoid-containing STT.

### Global features

Statistical analysis of global features showed that malignant myxoid-containing STTs had significantly higher kurtosis after adjustment for age, sex, and tumor volume (*P* = 0.040) when compared with benign myxoid-containing STTs. The *P*-value for kurtosis was not statistically significant before the adjustment. Other global features derived from the grey-level histogram, including mean ADC, were not significantly different between the two groups.

### Regional features

Regional features, which included both intensity variability and size-zone variability, were significantly higher in malignant myxoid-containing STTs before adjusting for age, sex, and tumor volume (*P =* 0.026 and 0.011). However, neither of the regional parameters were significantly different between the two groups after adjustment (*P* = 0.548 and 0.258).

### Local features

Local feature analysis showed that malignant myxoid-containing STTs had significantly higher energy (*P* = 0.034), correlation (*P*<0.001), and homogeneity (*P* = 0.003), and lower contrast (*P*<0.001) and variance (*P =* 0.001) when compared to benign myxoid-containing STTs after adjusting for age, sex, and tumor volume. Energy was not statistically significant, while correlation (*P* = 0.005), contrast (*P*<0.001), homogeneity (*P*<0.001) and variance (*P* = 0.006) were statistically significant before adjusting for age, sex, and tumor volume.

### ROC analyses

The diagnostic performance of texture parameters that showed significant difference between benign and malignant myxoid-containing STTs in ANCOVA are summarized in Table 4. Contrast showed excellent discriminatory power with the highest AUC (0.923, 0.794–0.983 of 95% CI; *P*<0.001), sensitivity, and specificity. The optimal cut-off value was 57.199, and using this value, the sensitivity and specificity were 94.12% and 86.96%, respectively. According to AUC value, correlation (0.884, 0.743–0.963 of 95% CI; *P*<0.001) and homogeneity (0.847, 0.698–0.941 of 95% CI; *P*<0.001) showed good diagnostic performance, and variance (0.752, 0.590–0.875 of 95% CI; *P =* 0.001) showed fair diagnostic performance. AUC of kurtosis (0.616, 0.449–0.765 of 95% CI; P = 0.198) and energy (0.575, 0.409–0.730 of 95% CI; P = 0.419) showed lower bound of 95% CI less than 0.5.

**Table 4:** Diagnostic performance of texture parameters for differentiating benign and malignant myxoid-containing soft tissue tumors.

Parameters	AUC (95% CI)	Optimal Threshold	Sensitivity (%)	Specificity (%)	*P*-value
Kurtosis	0.616 (0.449–0.765)	>3.151	70.59	52.17	0.198
Energy	0.575 (0.409–0.730)	>0.002	88.24	30.43	0.419
Correlation	0.884 (0.743–0.963)	>0.008	82.35	82.61	<0.001*
Homogeneity	0.847 (0.698–0.941)	>0.285	88.24	78.26	<0.001*
Contrast	0.923 (0.794–0.983)	≤57.199	94.12	86.96	<0.001*
Variance	0.752 (0.590–0.875)	≤76.979	88.24	52.17	0.001*

AUC = area under the curve

*P<0.05, statistically significant.

**Fig 4:**
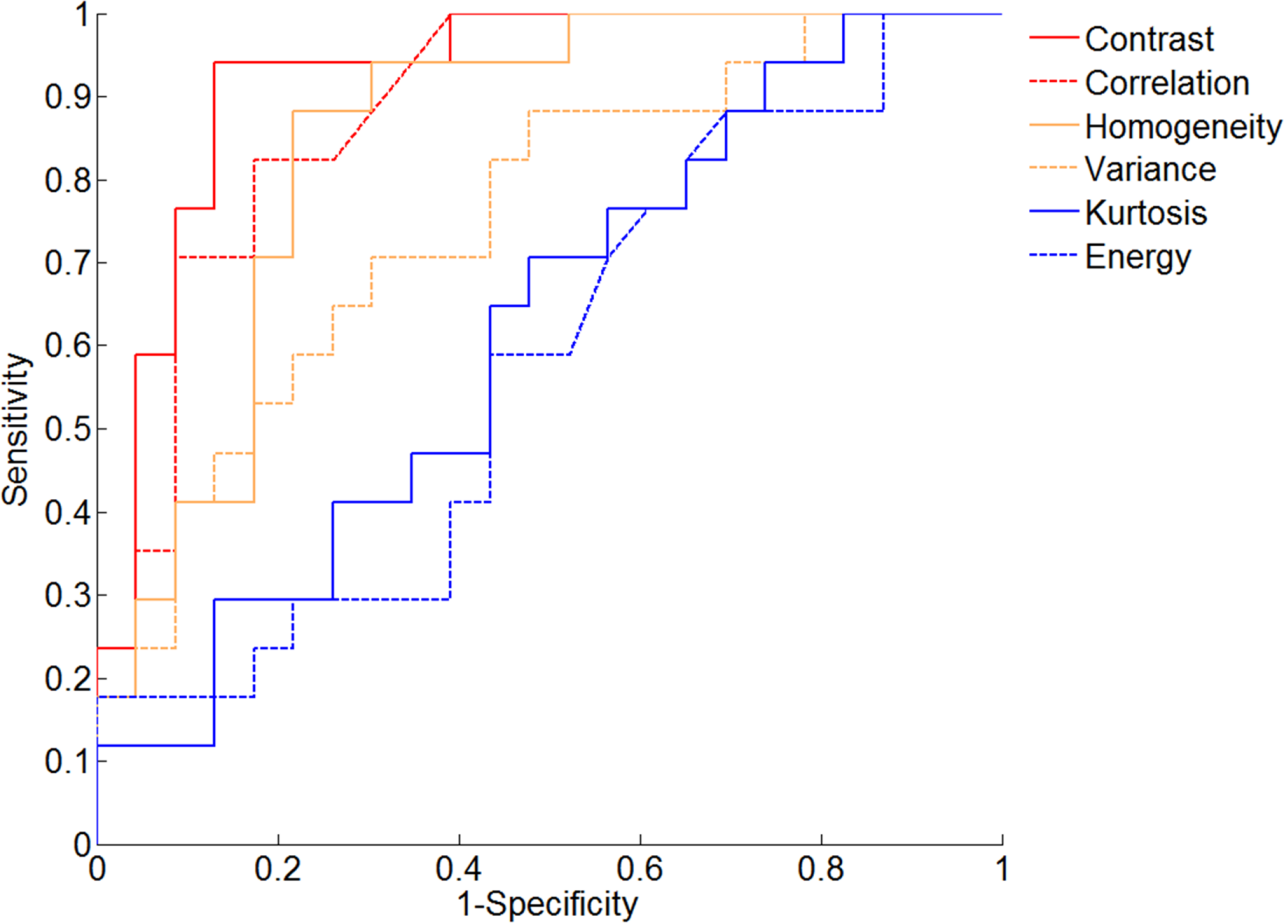
ROC curves of texture parameters for differentiating benign and malignant myxoid soft tissue tumors. The corresponding AUC values are listed in Table 4. Contrast showed the highest AUC (0.923, 0.794–0.983 of 95% CI; *P*<0.001) among the analyzed texture parameters.

Pairwise comparison showed that AUC of contrast for differentiating benign and malignant myxoid-containing STTs is significantly higher than those of other parameters except that of correlation (Table 5, Fig 4). AUC of homogeneity and variance were significantly higher than those of kurtosis and energy, respectively.

**Table 5:** Pairwise comparison of AUC value of texture parameters.

Parameters	vs	Difference between areas (95% CI)	*P*-value
Contrast	Correlation	0.040 (-0.010–0.089)	0.118
	Homogeneity	0.077 (0.006–0.147)	0.033*
	Variance	0.171 (0.051–0.292)	0.005*
	Energy	0.348 (0.190–0.505)	<0.001*
	Kurtosis	0.307 (0.158–0.456)	<0.001*
Correlation	Homogeneity	0.037 (-0.050–0.124)	0.402
	Variance	0.132 (0.035–0.228)	0.007*
	Energy	0.308 (0.152–0.464)	<0.001*
	Kurtosis	0.267 (0.128–0.406)	<0.001*
Homogeneity	Variance	0.095 (-0.049–0.238)	0.196
	Energy	0.271 (0.109–0.434)	0.001*
	Kurtosis	0.230 (0.066–0.394)	0.006*
Variance	Energy	0.176 (0.043–0.310)	0.004*
	Kurtosis	0.136 (0.017–0.254)	0.025*
Energy	Kurtosis	0.041 (-0.070–0.152)	0.469

*P<0.05, statistically significant.

## Discussion

Differentiation of benign and malignant myxoid-containing STTs based on imaging findings are often challenging for radiologists due to significant overlap of radiologic findings between the two on both conventional and functional MR sequences. Final diagnosis usually relies on pathologic evaluation of tissue specimen, but biopsy may not be routinely performed for all tumors in clinical practice. Thus there has been a need for novel imaging biomarker that can help differentiate benign and malignant myxoid-containing STTs.

In the current study, texture analysis based on ADC map was performed to examine the spatial heterogeneity of malignant and benign myxoid-containing STTs. We found significant difference between benign and malignant myxoid-containing STTs with regard to kurtosis computed from the histogram, and the energy, correlation, homogeneity, contrast, and variance computed from GLCM. As shown in Figs 2 and 3, the spatial pattern of the benign myxoid-containing STT is visually more heterogeneous than that of the malignant myxoid-containing STT on the ADC map. The significant difference of quantified parameters obtained through texture analysis in our result is reflection of this difference of spatial pattern on ADC map. An important finding of our study is that a number of ADC texture analysis parameters can reveal the difference between two tumor groups which were not demonstrated in comparison of mean ADC value.

Regarding local features, our results showed that malignant myxoid-containing STTs had significantly higher energy, correlation, and homogeneity, but significantly lower contrast and variance when compared to those of benign myxoid-containing STTs. Energy refers to a degree of non-uniformity, while correlation refers to a degree of linearity. Furthermore, homogeneity is defined as a degree of closeness in the distributed GLCM values. Contrast refers to a degree of difference in the neighboring values, and variance refers to how far each value is from the mean in GLCM. Our local texture analysis result revealed that malignant myxoid-containing STTs to be non-uniform, linear, clustered, similar, and narrower in distribution in GLCM when compared to benign myxoid-containing STTs. This result can be interpreted as malignant myxoid-containing STTs having a more spatially homogenous ADC map. ROC analyses revealed contrast, correlation, homogeneity, and variance to show overall good diagnostic performance. Especially, contrast showed excellent discriminatory power with the highest AUC value, sensitivity, and specificity. These promising results of local feature warrants further study for clinical application of theses texture parameters.

Global feature also revealed spatial pattern on the ADC maps of benign and malignant myxoid-containing STTs that were similar to those demonstrated for local feature. Malignant myxoid-containing STTs had significantly higher kurtosis after adjusting for age, sex, and tumor volume when compared with benign myxoid-containing STTs. A higher kurtosis indicates a sharp peak in the distribution of pixel values, which generally indicates a greater homogeneity of pixel values. In accordance with previously reported studies [8,25], there was no significant difference in mean ADC value between benign and malignant myxoid-containing STTs.

Previous studies using texture analysis of DWI showed significantly higher entropy in malignant tumors compared with those in benign tumors, suggesting that there is higher spatial heterogeneity in malignant tumors [12,22]. However, in this study, we found that malignant myxoid-containing STTs to be more spatially homogenous on ADC maps (higher kurtosis, energy, correlation, homogeneity, and lower contrast, variance in malignant myxoid-containing STTs). Our findings, which are contrary to those of previous studies, may be due to the underlying histologic characteristics of myxoid-containing tumors. Unlike non-myxoid tumors, the abundant extracellular matrix is a major textural component of myxoid-containing STTs [25,26]. On T1-weighted image, schwannomas are reported to demonstrate inhomogeneous signal and myxofibrosarcoma are reported to demonstrate homogeneous signal [20]. However, little has been reported on degree of signal homogeneity in myxoid-containing STTs on DWI. The majority (20/23, 87%) of benign myxoid-containing STTs in our data set were neurogenic tumors, which are reported to have a characteristic fibrous center with a myxoid periphery [27,28]. This histologic composition may have contributed to increased heterogeneity in diffusivity of benign myxoid-containing STTs. Further studies evaluating the difference in diffusivity between benign and malignant myxoid-containing STTs at cellular level, including the interplay with extracellular matrix, should be performed.

Regional features, such as intensity variability, and size-zone variability, were computed from GLSZM [16]. For regional texture analysis, the ADC values within the ROI were converted into 64 different values ranging from 1 to 64 (2^6^). In this study, GLSZM’s (m, n) was defined by the number of homogenous clusters, given the tumor size of the clusters (n, maximum: the number of voxels within ROI) to their clusters’ discrete values (m, maximum: 64). The size-zone variability was computed by emphasizing the GLSZM in terms of the tumor size (n), while the intensity variability was calculated by emphasizing the GLSZM in terms of the discrete values (m). Therefore, the row of GLSZM increases as the number of voxels within the ROI (tumor size) increases, leading to high size-zone variability for a large tumor ROI. Statistical analysis showed that size-zone variability was a good texture parameter to differentiate between malignant and benign myxoid-containing STTs; however, we could not find any statistical significance in size-zone variability after adjusting for age, sex, and tumor volume (*P* = 0.258).

Besides the intrinsic limits of a retrospective study, there were several limitations in our work. First, the study population was relatively small with a limited number of histologic subtypes. In addition, we did not compare the diagnostic accuracy of texture analysis parameters with that of conventional MRI parameters. Additionally, we did not perform histologic correlation to clarify the discrepancies in texture analysis parameters between the two groups. Lastly, image data analyses were done by a single reader.

In conclusion, we found that the spatial patterns on the ADC map in malignant myxoid-containing STTs are more homogenous than those in the benign myxoid-containing STTs, which is contrary to the previous studies mainly using non-myxoid tumors in other organs. ROC analyses revealed overall good diagnostic performance of local features suggesting the potential use of DWI texture analysis to classify benign and malignant myxoid-containing STTs. Further research on the association between tumor texture and underlying biological factors is needed.

## Supporting information

S1 Table Texture parameter analysis. (XLSX)
